# A Strengths-Based Approach to Increasing Nutrition Knowledge in Student-Athletes: The ‘Eat 2 Win’ Pilot Program

**DOI:** 10.3390/nu17020361

**Published:** 2025-01-20

**Authors:** Andrea Fuller, Stephen P. Bird

**Affiliations:** 1 School of Health and Medical Sciences, University of Southern Queensland, Ipswich 4305, Australia; andrea.fuller@unisq.edu.au; 2 Centre for Health Research, University of Southern Queensland, Ipswich 4305, Australia

**Keywords:** adolescent athletes, nutrition, energy, RED-S, low energy availability, carbohydrate, protein, hydration

## Abstract

**Background and Objectives**: Proper nutrition and hydration are essential for the health, growth, and athletic performance of student-athletes. Adequate energy availability and sufficient intake of macro- and micronutrients support adolescent development, prevent nutrient deficiencies, and reduce the risk of disordered eating. These challenges are particularly relevant to student-athletes, who are vulnerable to nutrition misinformation and often exhibit limited nutrition knowledge. This study aimed to evaluate the feasibility and acceptability of the ‘Eat 2 Win’ nutrition education pilot program for high school student-athletes and assess changes in nutrition knowledge using the Nutrition for Sport Knowledge Questionnaire—Adolescents (NSKQ-a). **Methods**: Fifty-five high school student-athletes (14.1 ± 2.2 years; 53% male, 47% female) from the Bremer State High School Sports Academy participated in the ‘Eat 2 Win’ program. The curriculum was tailored to align with the nutritional needs of Australian high school student-athletes and included three interactive workshops on sports nutrition concepts, practical cooking classes, and online learning modules. Sessions were delivered onsite at the school over three consecutive weeks, once per week. Twenty-four participants (43.6%) completed all program components. **Results**: The program was feasible and well-accepted by participants. Nutrition knowledge, assessed in 16 participants using the NSKQ-a, revealing an overall knowledge increase of 9.7%, with sub-category improvements ranging from 3.1% to 34.4%. **Conclusions**: The ‘Eat 2 Win’ pilot program improved student-athletes’ nutrition knowledge, particularly in macronutrients and hydration. Participant feedback highlighted enjoyment and positive impacts. Targeted nutrition education programs like ‘Eat 2 Win’ can empower student-athletes to make informed dietary choices by addressing knowledge gaps, debunking nutrition myths, and fostering positive dietary behaviours. Future programs should emphasize energy intake, practical skills, and accessible, actionable information.

## 1. Introduction

Adequate and appropriate nutrition and hydration are key to athlete health and performance. For the student-athlete, sufficient intake from a variety of foods is particularly crucial to support both the increased training and competition demands, and the increased requirements for macro- and micronutrients for growth and development [1,2]. Specifically, early adolescent nutrition plays a role in the timing of puberty, future adult height and bone mineral density, and body composition [3,4,5]. Inadequate energy intake in student-athletes has the potential to compromise body functions, with the associated nutrient-related deficiencies negatively impacting athletic performance [6], and with subsequent injury risk, as well as increasing the risk of chronic disease later in life [7,8]. Due to high levels of energy expenditure typical in student-athletes (well in excess of non-athletes), a common issue is inadequate energy intake [1]. The condition known as Relative Energy Deficiency in Sport (RED-S) results from Low Energy Availability (LEA) [6,8,9,10]. Energy availability is defined as the amount of energy left for the body’s physiological functions after the energy expended during exercise is subtracted from the energy intake [11]. Ironically, LEA often results in a decreased resting metabolic rate, and therefore a greater risk of higher body fat percentage, which is the opposite of what the individual restricting intake was aiming to achieve [10]. Longer term effects of RED-S include decreased cardiorespiratory fitness, neurodevelopment, and immune function [6,8].

**Figure 1:**
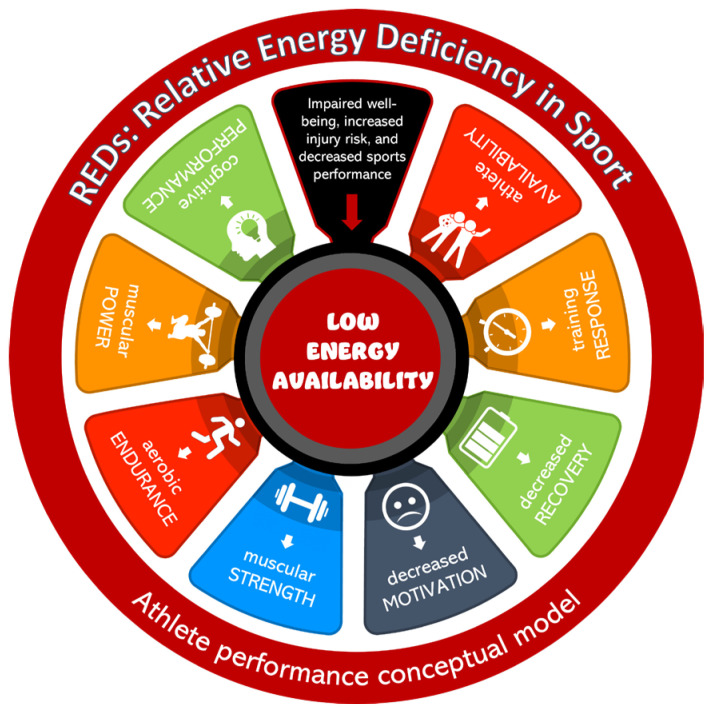
The REDs Athlete Performance Conceptual Model presents the effects of LEA on a continuum. LEA is associated with a variety of adverse REDs performance outcomes. Adapted from Mountjoy et al. [6].

Nutrition inadequacies are of significant concern during adolescence due to the increased energy requirements for growth and development, and the importance of maximising bone density during this life stage [11]. Female student-athletes are particularly at risk of LEA, which can result in delayed puberty, menstrual irregularities, poor bone health, disordered eating behaviours, and an increased injury risk [9]. Male student-athletes are also at risk of LEA, most commonly road cyclists and long-distance runners. Risks include the exercise-hypogonadal male condition, which results in lowered testosterone and libido [9]. Athletes in aesthetic sports and athletic events that emphasise body shape or leanness such as ballet, gymnastics, athletics, and sports with weight categories, are at higher risk of disordered eating behaviours, and thus at higher risk of LEA and RED-S [10,12]. Inadequate energy intake is often associated with disordered eating behaviours such as deliberately skipping meals, eliminating food groups, or attempting ‘fad’ diets. Nutritional risks associated with disordered eating, in addition to LEA, include macronutrient and micronutrient deficiencies, dehydration, electrolyte imbalances, and the risk of progression to a diagnosed eating disorder [10]. Collectively, these deficiencies are also associated with a decrease in athletic performance, including strength, power, endurance, and issues with managing fatigue, thermoregulation, and decision-making. Mountjoy and colleagues [6] term this the ‘REDs Athlete Performance Conceptual Model’ (Figure 1).

Inadequate energy intake often leads to student-athletes under-consuming foods high in key micronutrients. Micronutrients of particular concern are calcium, iron, and vitamin D [1]. Calcium, in conjunction with vitamin D, in adolescence is crucial for maximizing bone mineral density [13] and reducing the risk of stress fractures [14]. In some parts of Australia and globally, vitamin D may appear to be of less concern due to the predominately sunny climate [15]. However, due to the ‘SunSmart’ awareness campaign related to excessive sun exposure and skin cancer risk, vitamin D intake may be inadequate for certain individuals, particularly for those primarily training or competing indoors [16,17]. Iron deficiency is also a global concern [18], and student-athletes, particularly females, are at an increased risk due to iron losses in sweat and blood [19,20]. The consequences of inadequate iron intake on sporting performance include reduced endurance, increased muscle fatigue, increased lactate concentration, and the depletion of muscle glycogen [20].

A lack of nutrition knowledge has been consistently identified as a primary factor influencing the suboptimal dietary choices of student-athletes [21,22], which can negatively affect their overall health, well-being, and athletic performance. Therefore, the aims of the current study were to assess the nutrition knowledge of high school student-athletes; to describe the conceptualization, design, and implementation of the ‘Eat to Win’ pilot program—an innovative nutrition education initiative tailored for student-athletes within an Australian high school sports context; and to evaluate the program’s feasibility and acceptability. The ‘Eat 2 Win’ program was designed to address critical gaps in nutrition knowledge by identifying and incorporating essential components that support the growth and development of student-athletes, and thereby provide a strong foundation for informed and sustainable dietary behaviours.

## 2. Nutrition Knowledge

**Figure 2:**
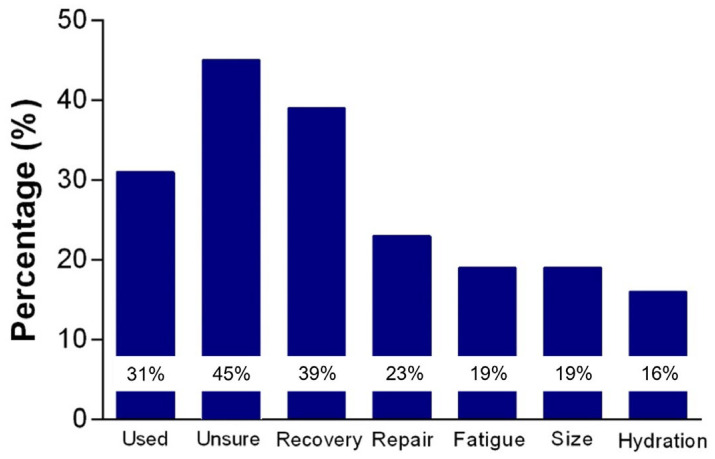
The percentage of youth academy athletes reporting protein supplement use in the previous 12 months and the reason provided for usage. Adapted from Bird and Rushton [21].

A primary reason why student-athletes make poor nutrition choices, including inadequate food and fluid intake, as well as inappropriate nutritional supplement usage, appears to be a lack of nutrition knowledge [21,22]. Student-athletes are often unaware of the importance of adequate energy intake in general and carbohydrate intake in particular. In addition to inadequate energy intake generally, student-athletes under-consume carbohydrates, while over-consuming protein [1], with excessive protein from common supplements [21,23]. They often underestimate their hydration needs, especially during prolonged or intense exercise, or in hot and humid conditions, being unaware of the link between dehydration and reduced physical and cognitive performance [20]. A recent systematic review of 32 studies found general and sports nutrition knowledge were lacking in adolescent athletes [22]. However, the authors highlighted that many of the studies used inadequately validated questionnaires, with mean nutrition knowledge scores varying from 33% to 91% [22]. Fourteen studies assessed hydration knowledge, and the number of correct scores in this domain varied between 33% and 88% [22]. Despite this variation in the results across studies, a common theme was poor knowledge around nutrition supplements, with most study participants answering ‘incorrect’ or ‘don’t know’ to those questions. For example, Bird and Rushton [21] surveyed 101 youth academy athletes aged 13–18 years, with 42% answering ‘unsure’ to questions related to the supplement subcategory. Notably, 31% reported using protein supplements (Figure 2); however, there was much uncertainty when asked why they were taking protein supplements, as 45% reported ‘unsure’. Overall, the mean ‘correct’ score for nutrition knowledge was 44%. In agreement with previous research, the authors concluded that elite youth athletes lack fundamental nutritional knowledge [21].

Jovanov and colleagues [23] examined 348 athletes aged 15–18 years and reported very high supplement use, particularly protein supplements, with the main motivation being the enhancement of athletic performance, but 72% of the participants were unaware of the health risks associated with supplements in general. Similar results regarding protein supplement knowledge and use were reported in an Australian study of 87 student-athletes aged 13–18 years [24]. Sixty percent of the participants used protein supplements; the main reason for consumption was to aid muscle recovery, with general performance enhancement also a motivator [24]. Despite the heterogeneity of nutrition knowledge tools and methodological issues highlighted in the systematic review by Hulland et al. [22], it is evident that there are common misconceptions surrounding protein supplementation, and misunderstandings about hydration and energy requirements. Additionally, there also appears to be a mistaken belief by student-athletes that consuming excessive amounts of protein will significantly enhance muscle growth and recovery. While protein is essential, balance with other macronutrients is also crucial, and excessive protein intake can lead to the neglect of other important nutrients.

Furthermore, many adolescent athletes also underestimate their hydration needs, especially during prolonged or intense exercise [25], and fail to adjust their food intake to meet the demands of training [26]. Such issues surrounding nutrition misinformation are exacerbated in student-athletes due to their susceptibility to messaging from coaches and social media influencers with limited nutrition knowledge [21]. Added to this are targeted student-athlete marketing campaigns from sports food, beverage, and supplement companies with nutrition messages that may undermine appropriate nutrition choices [1]. Nutrition messages that focusses on dietary and supplement strategies aimed at manipulating weight and body composition are particularly insidious [1,10], especially when devoid of an underlying ‘food first’ philosophy [27]. Close et al. [28] suggest that ‘where practically possible, nutrient provision should come from whole foods and drinks rather than from isolated food components, dietary supplements or sports foods’ (p. 371). This is a significant consideration, as attitudes toward food choices, nutritional supplements, and eating behaviours established during childhood and adolescence often persist into adulthood [29].

## 3. Nutrition Education

There is evidence that nutrition education programs can support student-athletes to make informed food choices [30,31,32] by increasing nutrition knowledge in general and addressing specific knowledge gaps [33]. A systematic review by Tam et al. [34] examined thirty-two nutrition education interventions delivered to athletes (mean age 17.4 years, 66.1% female; 56.3% university-level athletes; 75% based in the US) and found a significant increase in knowledge overall. However, due to the wide range of tools used to measure nutrition knowledge, it is difficult to compare outcomes between studies, and determine which delivery mode may be most effective. Additionally, a systematic review of nutrition education programs in athletes by Boidin et al. [35] compared dietary intake outcomes from interventions with a variety of education modalities that included face-to-face group lectures or workshops, or individual counselling. While more than half of the studies (12/22) reported a significant change in at least one nutrition parameter, it was again not possible to determine the overall impact of nutrition education on either knowledge or nutrition behaviours due to methodological differences in the way the nutrition education was delivered. Collectively, the authors noted limited research on the effectiveness of nutrition education interventions for athlete populations. This is an interesting observation given the substantial resources dedicated to collegiate nutrition programs [36], so there is a pressing need for well-designed, rigorous studies to guide and enhance future best practices [35].

### ‘Eat 2 Win’ Nutrition Education Program

**Figure 3:**
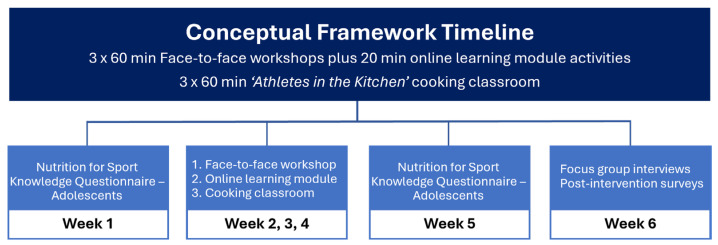
‘Eat 2 Win’ conceptual framework timeline.

The ‘Eat 2 Win’ student-athlete nutrition education program was developed by the University of Southern Queensland (Australia) using a multidisciplinary approach. The development team comprised academic staff from the School of Health and Medical Sciences, including an Accredited Practicing Dietitian, a Registered Nutritionist, an Accredited Exercise Physiologist, a strength and conditioning lecturer, and an educational designer, along with representatives from local sports high schools. Anecdotal evidence presented by stakeholders highlighted low nutrition knowledge among high school student-athletes across the region. It was suggested that gaps in nutrition knowledge resulted in poor dietary choices, including skipping meals, particularly breakfast, and/or training without proper nutrition or hydration. This descriptive feasibility pilot study aimed to (1) assess the acceptability and feasibility of the ‘Eat 2 Win’ program in sports high school student-athletes via feedback surveys and focus groups; and (2) to quantify student-athlete nutrition knowledge pre- and post-intervention using the Nutrition for Sport Knowledge Questionnaire—Adolescents (NSKQ-a). The ‘Eat 2 Win’ pilot was co-designed with the participating sports high school to align with the timeline constraints provided by the school and delivered through a combination of face-to-face nutrition education workshops, interactive online consolidation activities, and practical ‘Athletes in the Kitchen’ cooking classes. Figure 3 presents the conceptual framework timeline. Two key considerations were emphasised during the curriculum development phase: (1) the educational content is framed within a health and performance context to ensure that learning outcomes resonate with high school student-athletes; and (2) participants take away practical information that they can apply to their own dietary practices.

## 4. Materials and Methods

### 4.1. Participants

A total of 55 high school student-athletes from the Bremer State High School Sports Academy (Ipswich QLD, Australia) were enrolled in the ‘Eat 2 Win’ program (14.1 ± 2.2 years, 53% male, 47% female). The pilot was conducted onsite at the sports high school over three consecutive weeks, with sessions held once a week, delivered in two groups of 24 students (Group 1—Mondays, and Group 2—Thursdays). Students had the flexibility to attend either group session based on their school attendance and sport competition schedules, with 24 students (43.6%) attending all three sessions. Written approval was gained from the high school and the State Department of Education. The research was approved by the University of Southern Queensland Human Research Ethics Committee (ETH2024-0527). The findings are presented narratively with post-intervention focus group interview themes and written survey feedback supported by descriptive statistics.

### 4.2. Nutrition for Sport Knowledge Questionnaire—Adolescents (NSKQ-a)

The recently developed Nutrition for Sport Knowledge Questionnaire—Adolescents (NSKQ-a), adapted from the Abridged Nutrition for Sport Knowledge Questionnaire by Trakman et al. [37], has 29 items and takes ~10 min to complete. As per previous versions [37,38], the raw scores are converted into a percentage score with overall knowledge categorised according to the following score criteria: ‘poor’ knowledge (0–49%), ‘average’ knowledge (50–65%), ‘good’ knowledge (66–75%), and ‘excellent’ knowledge (75–100%).

### 4.3. Curriculum Design

The ‘Eat to Win’ curriculum was adapted from the Peak Health and Performance nutrition education program developed by Michigan State University [39] to suit Australian high school student-athlete cohorts. Specifically, the content was aligned to the Sports Dietitians Australia Position Statement on Sports Nutrition for the Adolescent Athlete [1]. Three nutrition education themes were identified: (1) adequate energy and carbohydrate intake for performance, (2) protein and carbohydrate intake for post-exercise recovery, and (3) hydration for performance and recovery. Furthermore, ‘Eat 2 Win’ was designed to provide practical dietary advice in the context of promoting nutritional habits that meet athletes’ training and performance goals and overall health, while dispelling myths around carbohydrates, protein, and nutritional supplements. The program emphasised a ‘food first’ [27] approach, which is particularly important during adolescence in establishing positive eating behaviours as a foundation for their future relationship with food [1,35].

#### 4.3.1. Nutrition Education Workshops

The ‘Eat 2 Win’ pilot consisted of three education modules (Table 1). Module 1 ‘Winning Nutrition’ provided general nutrition information covering fundamental concepts related to nutrition and fuelling for performance. Module 2 ‘Fuelling Performance’ specifically focused on sports nutrition, the importance of breakfast, and the ‘athlete’s plate’ [40]. Module 3 ‘Hydrate to Dominate’ covered hydration and fluid requirements, drink choices, and the signs and symptoms of dehydration. This module also outlined the ‘food first’ approach [27,28], and under what specific circumstances nutritional supplementation could be considered. All nutrition education workshops were delivered within 40 min school period blocks. Workshop facilitators noted that students would often lose concentration after ~30 min. Post-intervention survey student feedback confirmed that shorter education workshops would be better suited with delivery focused on key messages.

#### 4.3.2. ‘Athletes in the Kitchen’ Cooking Classroom

Our participants came from diverse family backgrounds, encompassing a broad spectrum of education levels and household incomes. Anecdotal evidence from school staff identified food insecurity as a potential issue for some students. As such, the recipes used in the cooking sessions were deliberately chosen as high-nutrient, low-cost options, in addition to being quick and easy to prepare. The cooking classes were very well received, with all post-intervention survey responses highlighting that taking part in cooking was the favourite part of the program and that it was fun cooking with their friends. Focus group feedback confirmed that the kitchen setup of 4-students per workstation was ideal. Everyone was able to be involved in food preparation, cooking, and clean-up. Some students reported having never cooked before or were nervous about taking part; however, they later commented how much they enjoyed being in the kitchen and eating the food they had cooked.

#### 4.3.3. Online Learning Module Activities

To complement the face-to-face workshop sessions, ‘Eat 2 Win’ incorporated 20 min online learning module activities through the University of Southern Queensland online portal (StudyDesk). This approach provided students with access to technology-enhanced resources, interactive learning activities, and peer-group discussion forums. While the intention was for students to consolidate their learning through the completion of the online learning activities in their own time, low engagement with the online portal was observed. Feedback from students suggested that dedicating time within the face-to-face workshop sessions to complete the online learning modules would be beneficial. This approach would allow students to receive immediate support from facilitators, collaborate with peers, and better link the online material with the hands-on cooking classes. Embedding the online learning modules into the workshop structure will promote student engagement and their comprehension of the material would significantly improve.

## 5. Results

### 5.1. Feasibility and Acceptability

The initial analysis of the feasibility and acceptability of the ‘Eat 2 Win’ program is positive. The feasibility and acceptability drivers of successful delivery in a sports high school setting link directly to key stakeholder engagement (i.e., school principal and staff) and enthusiasm about the program benefits for students. Feasibility is significantly increased when there is a high level of communication and collaboration between high school staff and the program team to: (1) manage the program timetable within school hours, and (2) facilitate suitable teaching space (i.e., workshop classroom and kitchen, including setup time prior to ‘Athletes in the Kitchen’). For the current pilot, workshops and cooking classes were delivered back-to-back, onsite at the school; however, future programs should consider different delivery time options or offsite locations for the cooking classes. There was a high level of acceptability by the participants. Post-intervention satisfaction survey feedback and focus group interview transcripts were consistently positive, with focus group participants confirming that they would do the program again and recommend it to other students.

As with any educational pilot program, there were challenges and limitations in its implementation. During the initial planning phase, the program was designed to be delivered as a one-hour nutrition education workshop—incorporating opportunities for group discussion and interactive learning activities, then followed by a one-hour ‘Athletes in the Kitchen’ cooking class. However, logistical constraints, including school operational hours, timetables, and sporting commitments, necessitated adjustments to program delivery. Specifically, while Group 1 received the education workshop followed by the cooking class as intended, Group 2 experienced the sessions in reverse order, with the cooking class preceding the education workshop. Post-intervention survey feedback revealed a preference for scheduling the cooking class after the education workshop. However, logistical challenges arose in coordinating the cooking classes due to the limited availability of the school kitchen. A practical oversight was kitchen preparation and setup time. Sufficient time must be allocated to ensure kitchen preparation, including pre-measuring ingredients for six workstations, each accommodating four students. For the ‘Athletes in the Kitchen’ cooking classroom, where students prepared two recipes each week, a minimum of 45 min is required for setup prior to the session. Table 2 outlines the key themes identified from post-intervention focus group interviews and written survey feedback.

### 5.2. Nutrition Knowledge Score

The impact of the ‘Eat 2 Win’ pilot on high school student-athlete nutrition knowledge was evaluated on 16 student-athletes using the NSKQ-a, with results summarised in Table 3. The overall nutrition knowledge score increased from 42.2 ± 23% (pre) to 51.9 ± 25% (post), representing a 9.7% improvement. This is also consistent across nutrition sub-categories. Among the sub-categories, the greatest improvement was observed in hydration knowledge, which increased by 34.4% (from 37.5 ± 22% to 71.9 ± 31%). Supplementation knowledge also demonstrated an increase of 13.5% (from 26.0 ± 16% to 39.6 ± 28%). Macronutrient knowledge improved by 6.6%, increasing from 51.0 ± 18% to 57.6 ± 21%. In contrast, vitamins showed the smallest improvement (3.1%), with scores increasing from 12.5 ± 18% to 15.6 ± 13%. Overall, while the current findings of the ‘Eat 2 Win’ pilot highlight the effectiveness of the program in enhancing high school student-athletes’ understanding of fundamental nutritional concepts, the results are considerably lower than the threshold scores representative of “good” sports nutrition knowledge, as suggested by Trakman et al. [38] (see Section 4.2).

## 6. Discussion

Nutrition is a critical component of supporting the growth, development, and athletic performance of student-athletes. Despite this, adolescent athletes often prioritize physical training over nutritional practices, which are frequently neglected. This neglect is closely tied to consistently low levels of nutrition knowledge reported across studies. The findings from the ‘Eat 2 Win’ pilot program align with previous research, demonstrating that baseline nutrition knowledge among youth athletes is generally limited. For instance, the baseline score of 42.2% observed in this study is comparable to the 47% reported by Trakman et al. [37], and slightly lower than the 49.6% and 52.5% reported by Scanlon and Norton [41] and Bird and Rushton [21], respectively, among youth academy and development athletes. The post-program score of 51.9% in the current study is similar to our previous findings [21] and reflects a 9.7% improvement. This is consistent with other intervention-based studies, which reported a 10% increase in overall nutrition knowledge following targeted education in female college athletes [42]. A particularly notable outcome of the current study was the significant gain in hydration knowledge (+34.4%), a finding that contrasts with prior research, where improvements in hydration understanding were less pronounced [21]. This suggests that the ‘Eat 2 Win’ program effectively emphasized hydration as a priority topic. However, the minimal improvement observed in vitamin knowledge (+3.1%) highlights the persistent challenge of addressing complex topics like micronutrients in student-athlete populations.

The ‘Eat 2 Win’ pilot program was designed as an initial step toward addressing these gaps by providing a structured framework for nutrition education tailored to the unique needs of student-athletes. While the results highlight measurable improvements, particularly in hydration knowledge, the findings also underscore the need for further refinement to achieve the desired “good” threshold of nutrition knowledge across all categories, as defined by Trakman et al. [37]. These limitations are consistent with the nature of a pilot study and reflect the program’s early stage of development. To expand on the significance of this work, it is essential to situate these findings within the broader context of nutrition education interventions for youth athletes. For example, studies by Zinn et al. [43] and Walsh et al. [44] similarly reported modest improvements in nutrition knowledge following educational interventions, suggesting that incremental gains are a realistic outcome in this population. The emphasis on practical, actionable knowledge (such as hydration and macronutrients) may serve as a strength of the ‘Eat 2 Win’ program, given that these areas are more immediately relevant and applicable to student-athletes’ daily routines. Looking forward, further development of the ‘Eat 2 Win’ program must address knowledge gaps in complex areas like vitamins and supplementation, potentially through greater emphasis on innovative teaching strategies for enhancing complex creativity, such as combined interactive workshops and gamified learning strategies. This is an important consideration, with recent work by Lee [45] highlighting that ‘students considered that gamification could stimulate their motivation, attitudes, and interest in learning’ (p. 11). Additionally, longer-term interventions with follow-up assessments are required to evaluate the retention of knowledge and its translation into sustainable dietary behaviours.

## 7. Conclusions

Student-athletes face unique nutritional demands, placing them at an increased risk of inadequate dietary intake [27]. Achieving optimal health and performance requires a comprehensive approach to nutrition, including informed choices about meals, snacks, and beverages throughout the day and around training or competition [1]. Addressing nutrition knowledge and suboptimal dietary practices commonly observed in adolescents [33] through targeted education programs, such as ‘Eat 2 Win,’ has the potential to enhance nutritional knowledge and positively influence long-term dietary behaviours. By equipping student-athletes with both nutrition knowledge and cooking skills, such programs foster independence, empower informed decision-making, and lay the foundation for a positive relationship with food. The ‘Eat 2 Win’ pilot demonstrated measurable improvements in nutrition knowledge among high school student-athletes, particularly in macronutrients and hydration knowledge. While the findings reveal areas for further refinement, they underscore the potential of targeted education initiatives to address critical knowledge gaps and support the unique nutritional needs of student-athletes. The ‘Eat 2 Win’ program establishes a valuable foundation for future nutrition education research to optimise health and performance outcomes in student-athletes.

## Figures and Tables

**Table 1:** ‘Eat 2 Win’ nutrition education topics and learning objectives.

Topic	Learning Objectives
Module 1. Winning Nutrition	Understand fundamental concepts related to nutrition and nutrients. Outline the role of optimum fuelling for performance. Identify the five food groups, macronutrients, and their functions.
Module 2. Fuelling Performance	Understand why breakfast is the most important meal of the day. Outline what to eat before and after training. Identify how much protein you need.
Module 3. Hydrate to Dominate	Understand the importance of hydration for sports performance. Choose the best drink options and timing for hydration. Understand the ‘food first’ approach to sport nutrition and that nutritional supplements are only required in very specific situations.

**Table 2:** ‘Eat 2 Win’ post-intervention focus group interview themes and written survey feedback.

Focus Group Feedback Themes	Written Student Feedback
Nutrition education workshops	
Enjoyment: Enjoyed the workshop presentations. Learning: Learned interesting things about nutrition and hydration. Interaction: Workshops need to be more interactive.	*‘The presenter made it relatable with examples that made sense to us.’* *‘The stuff on hydration really helped me. I’ve started drinking more water, and I feel better during training.’* *‘Learning what to eat before and after training has been super helpful.’* *‘The bit about the colour of your pee and hydration was really interesting.’*
‘Athletes in the Kitchen’ cooking classes	
Enjoyment: Cooking was fun. Learning: Linked to education workshop content. Interaction: Group size just right.	*‘It was fun to cook together as a group. It made it more enjoyable, and we could help each other.’* *‘I liked getting the information first, so I knew why we were cooking what we were cooking.’* *‘Everyone had something to do, so no one was just standing around.’* *‘The cooking sessions were fun, and the nutrition tips were easy to follow. I think other students would like it.’*
Online module learning activities	
Enjoyment: Didn’t have time to do it. Learning: Complete in workshop time. Interaction: Low engagement outside the classroom.	*‘It would have helped if we had time during the sessions to do [the online modules], instead of on our own.’* *‘It would be cool to hear about athletes using these nutrition strategies, so we know it really works.’* *‘A cheat sheet with quick tips would be great, so I could look back at it when I need to.’* *‘I kept forgetting to do them.’*

**Table 3:** NSKQ-a overall and subsection scores (*n* = 16).

Category	% Score (PRE)	Ranking	% Score (POST)	Ranking	% Change
Overall score	42.2 ± 23	Poor	51.9 ± 25	Average	9.7
Macronutrient	51.0 ± 18	Average	57.6 ± 21	Average	6.6
Hydration	37.5 ± 22	Poor	71.9 ± 31	Good	34.4
Supplementation	26.0 ± 16	Poor	39.6 ± 28	Poor	13.5
Vitamins	12.5 ± 18	Poor	15.6 ± 13	Poor	3.1

Abbreviations: NSKQ-a = Nutrition for Sport Knowledge Questionnaire—Adolescents; % = percentage.

## Data Availability

The raw data supporting the conclusions of this article will be made available by the authors on request.
